# Biomarkers and Diagnostics Will Play Essential Roles in Advancing Innovative Contraception

**DOI:** 10.3389/fendo.2022.897139

**Published:** 2022-07-14

**Authors:** Christopher C. Lindsey, Daniel S. Johnston

**Affiliations:** ^1^ Contraception Research Branch, Eunice Kennedy Shriver National Institute of Child Health and Human Development, National Institutes of Health, Bethesda, MD, United States; ^2^ Gynecologic Health and Disease Branch, Eunice Kennedy Shriver National Institute of Child Health and Human Development, National Institutes of Health, Bethesda, MD, United States

**Keywords:** contraception, biomarker, diagnostic, regulatory, spermatogenesis

## Introduction

The field of contraception development has exciting new momentum. Novel, innovative approaches will expand the contraceptive options available to men and women. However, the associated innovations present fresh challenges to clinical development and regulatory approval. How can stakeholders best ensure that obstacles will be overcome so the new discoveries will move from bench to bedside?

Translational medicine came to prominence over 20 years ago and arose from the need to improve the transfer of biological knowledge into medical utility. Critical components of translational medicine are the formation and utilization of animal models relevant to the human condition, as well as the development of diagnostics and biomarkers which are integral to drug development by informing evidence-based preclinical and clinical decisions. This opinion piece discusses the current need for biomarkers and diagnostics in the development of contraceptives and suggests how the field can work together toward that goal.

### Development of Tools

Biomarkers and diagnostics are generally named in connection to their usage. These include, but are not limited to disease biomarkers, patient selection biomarkers, pharmacodynamic (PD) biomarkers, and diagnostics to understand chemical concentration. For example, disease biomarkers indicate potential disease (e.g., prostate specific antigen test for prostate cancer). Patient selection biomarkers are predictive of a given person’s response to a drug, guiding clinical use or potentially inclusion in a clinical trial (e.g., HER2 amplification for cancer). Disease and patient selection biomarkers, not typically used directly for contraception, are important for other therapeutic areas and clearly illustrate the application of such tools.

The PD biomarker is relevant to all drug development. It indicates the drug effect on the molecular target in an organism and is typically as proximal to the target modulation site as feasibly possible to minimize any indirect effects. If the target is an enzyme, the PD biomarker is usually the product of the enzyme, and PD activity is read as a change in the concentration of the enzymatic product. If the assay is appropriately sensitive, a small amount of target modulation will result in the significant change in the PD biomarker.

However, other factors also impact the selection of a PD biomarker, such as sample procurement. For example, if the target is a testis-specific enzyme and an invasive testicular biopsy is required for direct target activity measurement, obtaining that sample for clinical development is unlikely. In such cases, an indirect readout such as ejaculated sperm number, sperm morphology or sperm motility may be employed as an alternative.

Consider the potential use of a PD biomarker for the male contraceptive target Soluble Adenylyl Cyclase (sAC), encoded for by the ADCY10 gene. The enzyme converts ATP to cAMP: a requirement of sperm motility. Targeted deletions of ADCY10 in mice result in male infertility due to impaired sperm motility (1). Two infertile men have been identified with identical frameshift mutations leading to premature translation termination upstream of the nucleotide binding site in sAC (2). Importantly, treatment of sperm lacking sAC activity with a cell-permeable cAMP analogue increases sperm motility significantly. An excellent PD biomarker to evaluate a sAC inhibitor’s function is the measurement of cAMP levels in freshly ejaculated sperm prior to and following test subject exposure of a putative sAC inhibitor.

To fully interpret the results of PD biomarker analysis, one must understand the drug pharmacokinetics (PK) at the target site by measuring the drug concentration associated with the target site (e.g., plasma, rete testis fluid, ejaculate, vaginal cavity secretions). The relationship of these two parameters, a “PK/PD relationship,” is critical for determining whether the active pharmaceutical ingredient is modulating the target of the drug (indicated by the PD biomarker), and whether the active ingredient concentration required to induce the observed modification of the PD biomarker results in the desired clinical endpoint. Although the science of PK is broader than local drug concentration (it incorporates the absorption, distribution, metabolism, and excretion of drugs), in the context of the PK/PD relationship, ‘PK’ implies local drug concentration.

### Deciding What to Use in the Clinic

For effective use in translational research, the development and validation of biomarkers requires ample time and investment. Novel biomarkers require significant pre-clinical research and development to ascertain their suitability for the clinical environment. Analytical validation, particularly evaluation of the biomarker test’s precision and accuracy, is critical. In some cases, biomarkers could be developed for use beyond the clinic and become commercial companion diagnostics (e.g., patient selection biomarkers, disease biomarkers), defined as medical devices by the Food and Drug Administration (FDA).

Similar to constraints that may arise from sample acquisition, the cost/benefit ratio of developing a PD biomarker that is proximal to the target and the significant time and resources needed to develop and validate a clinical biomarker for testing may give reason for more distal PD biomarker strategies to be considered. For example, as discussed above with respect to sAC inhibition, cAMP quantitation in ejaculated sperm is an excellent PD biomarker; however, an indirect method for assessing sAC activity could be evaluating sperm motility. This evaluation may be sufficiently useful without incurring the time and cost of developing a validated clinical cAMP assay for sperm. Andrology laboratories associated with large medical institutions are trained in standardized sperm evaluation methods (e.g., World Health Organization Guidelines) (3). The assumption is that the observed decrease in sperm motility following exposure to a sAC inhibitor is the result of target modulation (inhibition of sAC leading to a decrease in intracellular sperm cAMP concentrations) ultimately leading to decreased sperm motility.

### A Changing Paradigm

Attempts to develop a male contraceptive *via* the suppression of intratesticular androgen concentration has a long and well documented history (4–8). Regulatory agencies have been aware of the mechanism of suppressing endogenous androgen production for more than 30 years. The biomarker of azoospermia and its use as a biomarker of clinical utility for contraception is accepted by regulatory agencies.

This is not true for potential male contraceptive products that function *via* non-hormonal methods. The development of male contraceptives directed against highly selective/specific mechanisms of action (e.g., single gene products such as sAC) will require thoughtful interaction with regulatory agencies to efficiently establish a comfort level for each new paradigm (e.g., inhibition of meiosis, perturbation of spermiogenesis, inhibition of capacitation, inhibition of sperm motility).

### Discussion

We have attempted to demonstrate that the changing paradigm in the field of contraceptive development will benefit from the development of biomarkers and diagnostics to facilitate the translation from preclinical to clinical research. These tools will also facilitate interactions with regulatory agencies and inform decision making during the clinical trial process. A key question is how to stimulate progress to this end?

The *Eunice Kennedy Shriver* National Institute of Child Health and Human Development (NICHD) has emphasized the importance of biomarker development for contraceptive research programs in recent years. Since 2017, Request for Applications (RFAs) for the Contraception Research Centers have emphasized the need to pursue these studies. Since 2019, the NICHD’s Biological Testing Facility (PAR-21-078) and Chemical Screening and Optimization Facility (PAR-19-261) have supported contraceptive development to execute service requests for the generation of data supporting novel contraception development, including biomarker data. Despite these calls and opportunities, applications including biomarker identification and validation have been lacking and biomarker research has not been incorporated into funded contraception development programs.

To stimulate focus more directly in this area, in 2021 the NICHD published RFA-HD-22-018 specifically to support the development of biomarkers and diagnostics for new contraceptive methods by small business. Awards are expected in 2022. This RFA was an important commitment and highlights the priority placed on biomarkers/diagnostics. Hopefully, the resulting research will directly support product development and serve as a ‘seed crystal’ for programs that have yet to include biomarkers/diagnostics in their product development strategies.

**How Do We Best Move Forward in the Field of Contraception?**

The field would benefit from the organization and execution of a focused workshop, highlighting late-stage preclinical contraception programs and prioritizing discussions about biomarker implementation. A critical component of such a workshop would be the active participation of key opinion leaders, industry representatives, and regulatory agency personnel (e.g., regulatory agency pharmacology/toxicology and clinical staff). Upon adjournment, timely communication of the workshop outcomes would ideally broadcast the key findings, next steps, and regulatory considerations, and thereby deepen information available to the field and strengthen the prospects of all worthy novel approaches.

**Could There be Broader Implications for Reproductive Biology/Health?**

We believe so. In recent years industry has shown renewed interest in the reproductive health space. Going forward, if a consortium involving industry as well as innovative product developers from academia and small business were to be established, enormous benefit could be realized. Reaching consensus on diagnostics and biomarkers and their validation would expedite product development. The cumulative impact would benefit not only the field of contraception, but all stakeholders across the spectrum of reproductive biology/health.

## Author Contributions

All authors listed have made a substantial, direct, and intellectual contribution to the work and approved it for publication.

## Funding

The authors are employees of the National Institutes of Health. *Comments and views of the authors do not necessarily represent the views of the NICHD, NIH, or federal government.*

## Conflict of Interest

The authors declare that the research was conducted in the absence of any commercial or financial relationships that could be construed as a potential conflict of interest.

## Publisher’s Note

All claims expressed in this article are solely those of the authors and do not necessarily represent those of their affiliated organizations, or those of the publisher, the editors and the reviewers. Any product that may be evaluated in this article, or claim that may be made by its manufacturer, is not guaranteed or endorsed by the publisher.
